# Intratumoral Administration of High-Concentration Nitric Oxide and Anti-mPD-1 Treatment Improves Tumor Regression Rates and Survival in CT26 Tumor-Bearing Mice

**DOI:** 10.3390/cells12202439

**Published:** 2023-10-11

**Authors:** Hila Confino, Yogev Sela, Yana Epshtein, Lidor Malka, Matan Goldshtein, Selena Chaisson, Steve Lisi, Amir Avniel, Jedidiah Mercer Monson, Frederick M. Dirbas

**Affiliations:** 1 Beyond Cancer, Rehovot 7608801, Israel; ysela@beyondcancer.com (Y.S.); yepshtein@beyondcancer.com (Y.E.); lmalka@beyondcancer.com (L.M.); mgoldshtein@beyondcancer.com (M.G.); 2 Beyond Cancer, Atlanta, GA 30305, USA; schaisson@beyondcancer.com (S.C.); jmonson@beyondcancer.com (J.M.M.); 3 Beyond Air, Garden City, NY 11530, USA; slisi@beyondair.net (S.L.); aavniel@beyondair.net (A.A.); 4 Beyond Air Inc., Rehovot 7608801, Israel; 5 California Cancer Associates for Research and Excellence, Fresno, CA 93720, USA; 6 Department of General Surgery, Stanford University, Stanford, CA 94304, USA; dirbas@stanford.edu

**Keywords:** gaseous nitric oxide, immune checkpoint inhibitors, solid tumors, metastasis, cancer immunotherapy

## Abstract

Background: Immune checkpoint inhibitors have transformed clinical oncology. However, their use is limited as response is observed in only ~20–50% of patients. Previously, we demonstrated that treating CT26 tumor-bearing mice with ultra-high-concentration gaseous nitric oxide (UNO) followed by tumor resection stimulated antitumor immune responses. Accordingly, UNO may improve tumor response to immune checkpoint inhibitors. Here, we investigated the ability of UNO to improve the efficacy of a programmed cell death protein-1 (PD-1) antibody in vitro and in treating CT26 tumor-bearing mice. Methods: CT26 cells were injected into the flank of Balb/c mice (*n* = 15–16 per group). On day 6, CT26 cells were injected into the contralateral flank, and anti-mPD-1 injections commenced. Primary tumors were treated with intratumoral UNO on day 8. Tumor volume, response rates, toxicity, and survival were monitored. Results: (1) Short exposure to 25,000–100,000 parts per million (ppm) UNO in vitro resulted in significant upregulation of PD-L1 expression on CT26 cells. (2) UNO treatment in vivo consistently reduced cell viability in CT26 tumors. (3) Treatment reduced regulatory T-cell (Treg) levels in the tumor and increased levels of systemic M1 macrophages. UNO responders had increased CD8+ T-cell tumor infiltration. (4) Nine days after treatment, primary tumor growth was significantly lower in the combination arm vs. anti-mPD-1 alone (*p* = 0.0005). (5) Complete tumor regression occurred in 8/15 (53%) of mice treated with a combination of 10 min UNO and anti-mPD-1, 100 days post-treatment, compared to 4/16 (25%) of controls treated with anti-mPD-1 alone (*p* = 0.1489). (6) There was no toxicity associated with UNO treatment. (7) Combination treatment showed a trend toward increased survival 100 days post-treatment compared to anti-mPD-1 alone (*p* = 0.0653). Conclusion: Combining high-concentration NO and immune checkpoint inhibitors warrants further assessment especially in tumors resistant to checkpoint inhibitor therapy.

## 1. Introduction

Immune checkpoint inhibitors (ICIs) have revolutionized the management of many cancers [1,2,3,4,5] by producing durable responses in patients with metastatic disease [6,7]. However, despite their remarkable clinical efficacy in some patients, ICIs are unable to improve tumor response in others and can also induce immune-related toxicities that resemble classic autoimmune diseases [1,7,8,9]. ICI resistance in a subset of patients limits the number of patients able to achieve long-lasting responses [2,10,11].

ICI blocks cancer cell-expressed receptors upregulated on immune cells [12]. PD-1 is an immune checkpoint protein on T-cells that binds to PD-L1/2, a ligand found on normal and cancer cell surfaces which provides a therapeutic target [13]. Upon binding, the T-cell receptor interaction with cancer cells and co-stimulatory signals are impaired [14,15]. Monoclonal antibodies directed against either PD-1 or PD-L1 can block the interaction of PD-1 and its ligands and enhance T-cell responses [15].

Hot, inflamed tumors have the greatest potential to respond to ICIs, while those lacking tumor-infiltrated T-cells are often resistant to ICIs [16,17]. For example, the mouse colon hot tumor model, CT26, a murine colorectal carcinoma and one of the most utilized murine solid tumor models, partially responds to ICI therapy [17]. More specifically, CT26 cells show statistically significant reductions in tumor volumes in response to murine anti-cytotoxic T-lymphocyte-associated protein-4 (CTLA-4) treatment and anti-PD-1 treatment [18]. Improved treatment response to ICI in a murine CT26 model could suggest improved treatment responses in humans.

Up to 50% of patients with PD-L1-positive tumors in humans show resistance or relapse after PD-1/PD-L1 inhibition [19] and the ability to accurately predict the response to immune checkpoint blockade is suboptimal [20] due to the complexity of establishing uniform predictive biomarkers [13]. For example, a high tumor mutation burden fails to predict the immune checkpoint blockade response [21], while PD-L1 expression alone, as a predictive biomarker, has limitations [13].

Several methods have been reported to predict a patient’s response to ICIs. For example, since the level of immune infiltration into the tumors strongly influences patient outcomes, assessment of infiltration has predictive value [10,22]. Another approach to predict the ICI response is by sequencing tumor mutations. Tumors harboring mutations in mismatch repair (MMR) genes have a reduced capacity to correct DNA replication errors, often resulting in microsatellite instability (MSI), making them sensitive to immune checkpoint blockade [20].

Several mechanisms may also result in PD-L1 upregulation. Pro-inflammatory cytokine release can augment PD-L1 expression [23]. In 2020, Kiriyama et al. proposed a mechanism for PD-L1 upregulation in A172 glioblastoma cells. In this study, the researchers revealed that NOC-18, an NO donor, increased expression of PD-L1 in A172 cells via the c-Jun N-terminal kinase pathway [24]. Radiotherapy and chemotherapy can also induce PD-L1 upregulation on cancer cells. In 2015, Peng et al. reported that chemotherapy induced an NF-kB-mediated PD-L1 upregulation in an ovarian cancer cell line [25]. In 2022, Wang uncovered the potential of radiotherapy to sensitize cancer cells to ICI therapy. Radiotherapy induces DNA breaks, resulting in a series of biological events that play an important role in immunomodulatory responses. PD-L1-upregulated expression on cancer cells occurs via four primary mechanisms: (I) the DNA damage signaling pathway; (II) interferon gamma (IFN-γ) signaling; (III) the cGAS-STING pathway; and (IV) the epidermal growth factor receptor (EGFR) pathway. All four of these mechanisms are involved in the JAK-STAT pathway [26].

The signaling molecule nitric oxide (NO) is crucial in cancer pathogenesis. At elevated concentrations, endogenous NO acts as an antitumor agent [27] and has been reported to sensitize resistant tumor cells to standard anti-cancer therapies, such as immunotherapy, chemotherapy, and radiotherapy [28,29]. Furthermore, NO exerts cytotoxic effects leading to immunogenic cell death (ICD), which is accompanied by the release of immunogenic damage-associated molecule patterns (DAMPs) to trigger a long-term protective antitumor response [30].

As previously published, we tested the efficacy of short-term intratumoral administration of 20,000 or 50,000 parts per million (ppm) UNO. Fourteen days after treatment when primary tumors were resected, the number of T-cells that penetrated the tumor was significantly elevated compared to the control treatment by immunohistochemical analysis. Twenty-one days post-UNO treatment, systemic T- and B-cells were elevated in the blood and spleen. The mice were then inoculated with a second dose of CT26 cells to the contralateral flank. Of the mice treated with 50,000 ppm UNO, 88.9% did not develop a secondary CT26 tumor at 21 days post-treatment [31].

Due to the potent immune response observed after treating primary tumors with UNO for 5 min, we hypothesized that combining anti-mPD-1, an ICI, with UNO could lead to a synergistic immune response, thus achieving broader and more durable responses than either agent alone.

## 2. Methods

### 2.1. UNO Gas

Between 25,000 and 100,000 ppm NO was administered from 2.9 L cylinders, with nitrogen (N2) as its stabilizing gas (Gordon Gas and Chemicals, Tel Aviv, Israel). All procedures were performed in a chemical hood. Gases were delivered via a pressure regulator through a PVC hose (International Biomedical, Austin, TX, USA). The flow rate was set to 0.2 L per minute (LPM) (for in vivo studies) and 1.0 LPM (for in vitro studies) using a manual flow meter. More comprehensive detail of the delivery system is available in Appendix A.

### 2.2. Tumor Cell Lines

Murine colorectal carcinoma cell line CT26 and murine triple negative breast cancer cell line 4T1 were grown in RPMI-based media (ATCC, Manassas, VA, USA), supplemented with 10% fetal bovine serum and 1% penicillin-streptomycin (Sartorius, Beit HaEmek, Israel). The human pancreatic adenocarcinoma cell line, Panc 02.03, was grown in RPMI-based media (ATCC) supplemented with 15% fetal bovine serum, 1% penicillin-streptomycin (Sartorius), and 10 units/mL human recombinant insulin (Merck, Rahway, NJ, USA). All cell lines were purchased from the American Type Culture Collection (ATCC) local distributor, Sartorius (Beit HaEmek, Israel).

### 2.3. Preparation of Tumor Cells

Tumor cell suspensions were prepared in a cell culture medium or Hanks’ Balanced Salt Solution (HBSS; Sartorius) at concentrations of 1 × 10^5^ cells/mL for in vitro studies or 5.0 × 10^6^ cells/mL for in vivo studies. Freshly prepared cells were grown to 70% confluency, harvested using trypsin (Sartorius), and counted using a hemocytometer.

### 2.4. In Vitro Studies

To assess cell viability after treatment with UNO, cells were seeded in 96-well plates at 10,000 cells per well and left to grow for 24 h. After 24 h, cell culture media were removed, and cells were exposed to UNO or N2 as the control for up to 1 min. Around 25,000–100,000 ppm UNO was delivered at 1.0 LPM in a 1.7 L box. Immediately following gas exposure, cell culture medium was restored and cells were incubated at 37 °C in 5% CO_2_ overnight. In preparation for cell analysis using flow cytometry, all media from each well were collected and cells were detached using trypsin. Cells and media were transferred to a microcentrifuge tube and centrifuged to maintain all cell populations. The supernatant was discarded, and further handling was performed according to the manufacturer’s instructions in the Annexin V-FITC kit (Cat. No. 130-092-052; Miltenyi Biotec, Bergisch Gladbach, Germany). Cell viability was assessed using Annexin V-Propidium Iodide staining. PD-L1 upregulation was assessed via staining with fluorescently labeled anti-mPD-L1 antibodies (Cat. No. 124315; BioLegend, San Diego, CA, USA) and flow cytometry analysis.

### 2.5. In Vivo Studies

CT26 cells were inoculated subcutaneously (s.c.) on the right flank of female and male Balb/c mice (8–10-week-old; Envigo, Israel) at a concentration of 5.0 × 10^5^ CT26 cells in 100 µL HBSS. Mice were evaluated for tumor volume using a digital caliper. Treatments were initiated when tumors reached an average volume of ~80 mm^3^ (usually eight days following tumor inoculation). More details regarding animal treatment are available in Appendix A.

#### 2.5.1. Immune Profiling and Measurement of Cell Viability In Vivo after UNO vs. Control

After tumors reached the desired target volume as noted above, tumors were infused with UNO or N2 for 5 min at 0.2 LPM or sham treatment (without gas administration) then euthanized 1, 5, or 7 days after this treatment. For each mouse, tumor and blood samples were extracted for analysis. Blood was drawn via intracardial puncture, and samples were treated with ACK buffer (Gibco, Billings, MT, USA) for 5 min at room temperature to eliminate red blood cells. Tumors were dissociated into single cells using gentleMACS (Miltenyi) and 0.2% collagenase/RPMI solution (both from Gibco). Cells were counted, and 1M cells were stained with the viable dyes Ghost Dye 710 (Tonbo, San Diego, CA, USA) or Zombie NIR (BioLegend) for 15 min, followed by immunostaining for T-regs and M1 macrophages markers (defined below) for 30 min. Staining for the T-cell panel was preceded by a 10’ Fc Receptor (FcR) block (Miltenyi) and utilized brilliant stain buffer (BD). Stained cells were fixed for 45 min with Foxp3 fixation/permeabilization (eBioscience, San Diego, CA, USA) and incubated with flow cytometry staining buffer (eBioscience) at 4 °C overnight. Following overnight incubation, cells were further permeabilized and stained intracellularly for 30 min, then washed, and data for both panels were acquired using a ZE5 flow cytometer. Data were then analyzed using FlowJO (BD, Franklin Lakes, NJ, USA). A complete list of the antibodies used is in Appendix A.

#### 2.5.2. Immune Profiling and Measurement of Cell Viability In Vivo after UNO + ICI

As noted above, but with the addition of anti-mPD-1: Up to 5 doses, for an overall cumulative dose of 25 mg/kg over nine days of anti-mPD-1 (RMP1-14 BP0146, LOT-810421N1; Bio X Cell, Lebanon, NH, USA), were injected i.p. every two days beginning two days before treatment with UNO.

For both UNO alone and UNO + ICI, visual and palpable observations were conducted to monitor the appearance of tumor recurrence in the contralateral flank or elsewhere 2 to 3 times per week.

### 2.6. Statistical Analysis

Statistical analyses were performed using Excel (Microsoft, Redmond, WA, USA) or GraphPad Prism 9.3.1 (GraphPad Software, San Diego, CA USA) with *p* < 0.05 considered statistically significant unless stated otherwise.

## 3. Results

### 3.1. PD-L1 Upregulation in CT26 and 4T1 Cells after In Vitro Exposure to UNO

**Figure 1:**
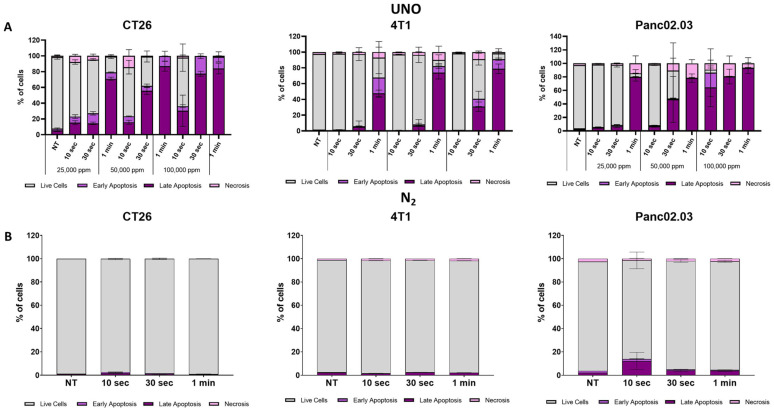
Short-term exposure of tumor cells to UNO induces apoptotic and non-apoptotic cell death. (**A**) CT26, 4T1, and Panc02.03 cell viability was analyzed using Annexin V-PI fluorescence analysis 24 h after exposure to 25,000, 50,000, and 100,000 ppm UNO for 10 s, 30 s, or 1 min or untreated. (**B**) Analysis of CT26, 4T1, and Panc02.03 cell viability after exposure to nitrogen. Data represent triplicates from a single experiment.

We first examined the effects of UNO on cancer cell viability in vitro (Figure 1A). CT26 cells were exposed to 25,000, 50,000, or 100,000 ppm UNO for 10 s, 30 s, or 1 min and cell viability was evaluated using Annexin V-PI staining 24 h post-exposure. Annexin V-PI cell death marker analysis showed UNO’s time- and dose-dependent effects on cell death. After 10 s of exposure to UNO, 23.0%, 23.4%, and 36.1% of CT26 treated with 25,000, 50,000, or 100,000 ppm gNO, respectively, were apoptotic (early + late apoptosis). After a 30 s exposure, Annexin V-PI cell death marker analysis revealed that 27.1%, 62.0%, and 99.6% of CT26 treated with 25,000, 50,000, or 100,000 ppm gNO, respectively, were apoptotic (early + late apoptosis). Following 1 min of exposure, 79.1%, 99.7%, and 98.8% of CT26 treated with 25,000, 50,000, or 100,000 ppm gNO, respectively, were apoptotic (early + late apoptosis). Similar results were observed in the murine triple negative breast cancer cell line, 4T1, and the human pancreatic adenocarcinoma cell line, Panc02.03. In contrast, exposing cells to nitrogen did not affect their viability (Figure 1B). Therefore, short exposure to UNO efficiently induces apoptotic cell death in tumor cells in vitro.

**Figure 2:**
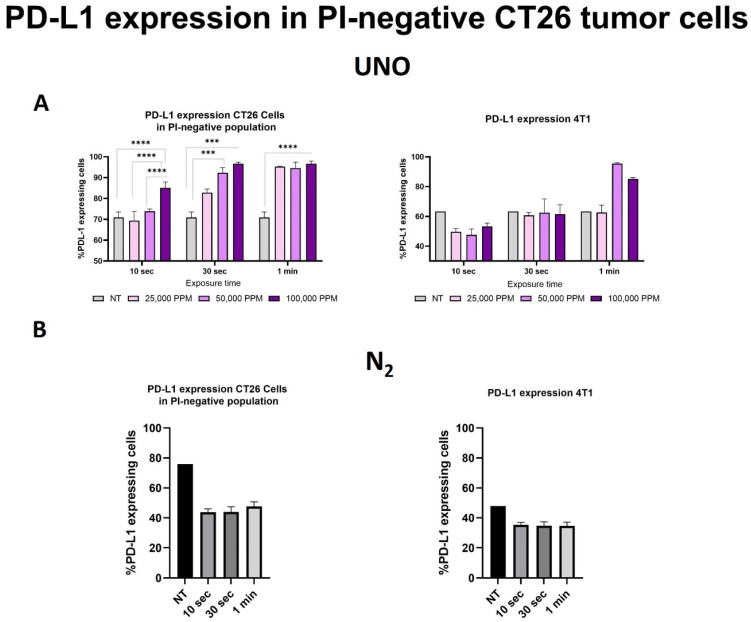
PD-L1 expression on CT26 and 4T1 cells 24 h after exposure to 25,000–100,000 ppm UNO. PD-L1 expression on CT26 and 4T1 cells was assessed by flow cytometry analysis using a labeled anti-PD-L1 antibody. (**A**) PD-L1 expression on CT-26 and 4T1 cells following exposure to UNO at different concentrations for different exposure times. (**B**) PD-L1 expression following N_2_ treatment for different exposure times. Data represent triplicates from a single experiment. NT-untreated two-way ANOVA, multiple comparisons test, α = 0.05, *** *p* < 0.001, **** *p* < 0.0001.

Next, we assessed PD-L1 expression 24 h after exposure to UNO on viable and early apoptotic CT26 cells (Figure 2A). Following 10 s of exposure to 100,000 ppm UNO, PD-L1 was expressed in 85.1% of the viable and early apoptotic cells, compared to 70.9% of untreated cells (*p* < 0.0001). Exposure to 25,000 or 50,000 ppm gNO for 10 s did not significantly change PD-L1 expression. However, exposure to 100,000 ppm NO for 30 s increased the percentage of PD-L1-expressing cells up to 96.7% (*p* < 0.0001 compared to untreated cells). Lower UNO concentrations (25,000 or 50,000 ppm) at this longer exposure time also increased PD-L1-expressing cells to 82.8% and 92.3%, respectively. Finally, a 1 min exposure of CT26 cells at all UNO concentrations induced PD-L1 expression in 94.6–96.6% of viable and early apoptotic CT26 cells, significantly higher than the 70.9% of cells that expressed PD-L1 under basal conditions (*p* < 0.0001). Importantly, PD-L1 expression did not increase upon exposure to N_2_ (Figure 2B) and was in fact lower compared to untreated cells. Similar results were observed in 4T1 cells albeit a higher threshold for PD-L1 upregulation (Figure 2A,B). These results show that short exposure of CT26 and 4T1 cells to UNO results in a dose-dependent upregulation of PD-L1 expression. This suggests that local treatment of solid tumors with UNO may sensitize “cold” tumor cells within the tumor mass to become responsive to immune checkpoint blockade and improve the efficacy of immune checkpoint blockade, due to upregulation of PDL-1 in the tumor microenvironment, as previously shown by Wu et al. [32]. Additionally, previously reported data with UNO treatment showed benefits of increased tumor infiltration and systemic response of several adaptive immune cells such as T-cells, dendrocytes, and B-cells which resulted in a significant reduction in the formation of challenge tumors and an improvement in mice survival [31].

### 3.2. UNO Reduces Cell Viability in CT26 Tumors and Potentiates Antitumor Immunity In Vivo

**Figure 3:**
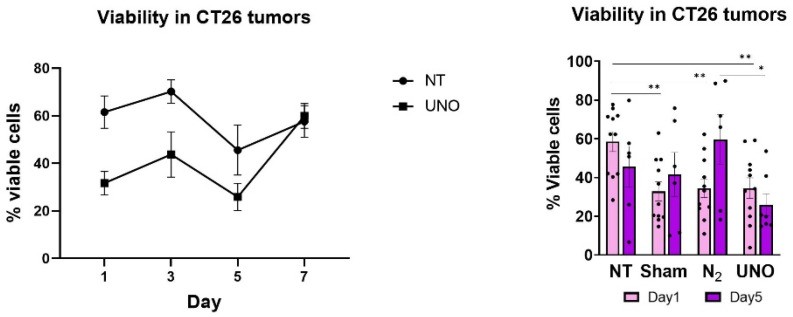
Cell viability in CT26 tumors treated with UNO. Cell viability in CT26 tumors post-treatment with 50,000 ppm UNO or N_2_ for 5 min was assessed by flow cytometry (Ghost Dye 710). Data were analyzed by one-way ANOVA multiple comparisons test, * *p* < 0.05, ** *p* < 0.01.

To determine whether UNO exerts cytotoxic pressure on tumor cells in vivo, we inoculated CT26 tumor cells into BALB/c mice and treated the tumors with 50,000 ppm gNO for 5 min. Control arms included N_2_ and sham treatment arms for 5 min, along with untreated mice. UNO administration resulted in reduced cell viability 1 day post-treatment, suggesting acute tissue damage attributed to the procedure (Figure 3). However, cell viability after 5 days remained low only in UNO-treated mice. In contrast, tumors of both N_2_- and sham-treated animals recovered, suggesting that UNO treatment induces persistent cytotoxicity in CT26 tumors.

**Figure 4:**
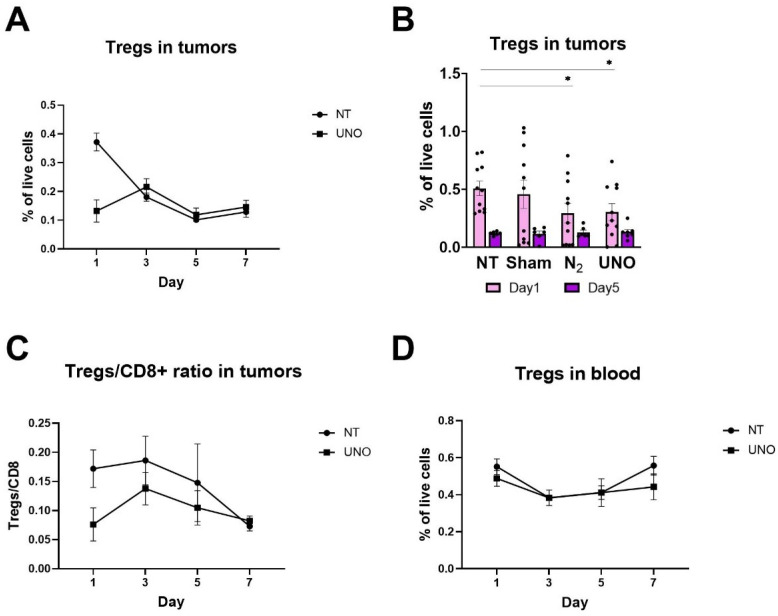
T-cell profiling in tumors and blood of CT26 tumor-bearing mice treated with UNO. (**A**,**B**) Levels of Tregs in CT26 tumors following UNO treatment. (**C**) Ratio of Tregs/CD8+ T-cells in CT26 tumors. (**D**) Levels of Tregs in the blood of CT26 tumor-bearing mice. Data were analyzed by one-way ANOVA multiple comparisons test, * *p* < 0.05.

Immunogenic cell death has been demonstrated to elicit an immune response by several mechanisms. To determine how UNO-mediated cell death affects tumor immunity, we performed immune profiling of tumors and blood from CT26 tumor-bearing mice taken 1 day post-UNO treatment. Evaluation of T-cell infiltration into CT26 tumors revealed significantly reduced Treg levels 1 day post-UNO treatment (Figure 4A,B). Furthermore, the ratio of Tregs/CD8+ was lower in UNO-treated mice one day post-treatment and remained lower 5 days post-UNO treatment (Figure 4C), indicating a favorable immune microenvironment after exposure to UNO (Figure 4D). Reduction in Tregs was also measured in the blood of CT26 tumor-bearing mice at day 7 post-treatment, possibly reflecting systemic changes over time (Figure 4D).

**Figure 5:**
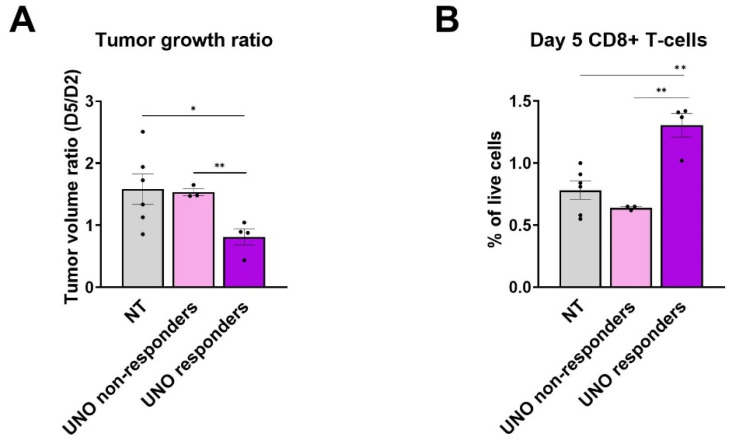
CD8+ T-cell infiltration into CT26 tumors in UNO responders and non-responders. (**A**) Volumetric tumor ratios on day 5 vs. day 2. (**B**) CD8+ T-cell tumor infiltration in UNO responders and non-responders on day 5. Data were analyzed by one-way ANOVA multiple comparisons test, * *p* < 0.05, ** *p* < 0.01.

We then determined the relationship between response to UNO treatment and T-cell infiltration to CT26 tumors. Mice displayed variable tumor growth following UNO treatment and could be divided into responders and non-responders (Figure 5A). Responders exhibited significantly higher CD8+ T-cell infiltration than non-responders and untreated mice on day 5 post-treatment, suggesting prolonged benefits in this subset of treated mice (Figure 5B).

**Figure 6:**
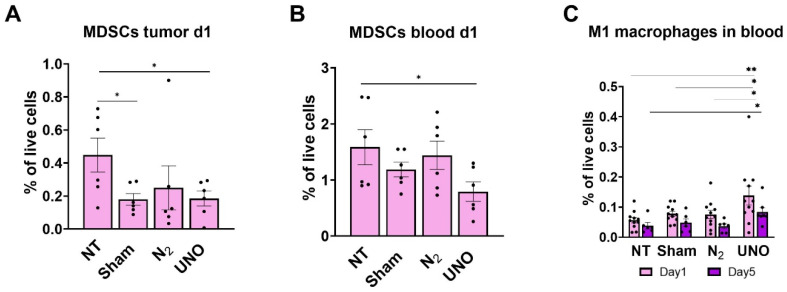
Myeloid cell profiling in CT26 tumors treated with UNO. Levels of mononuclear MDSCs (**A**) in tumors and (**B**) in blood of CT26 tumor-bearing mice. (**C**) Levels of M1 macrophages in the blood of CT26 tumor-bearing mice on day 1 and day 5 post-treatment. Data were analyzed by one-way ANOVA multiple comparisons test, * *p* < 0.05, ** *p* < 0.01.

We further explored the myeloid cells in the tumor microenvironment (TME) and blood following UNO treatment. In the TME, M-MDSC levels were reduced one day post-treatment with either UNO, sham, or N_2_ (Figure 6A). However, M-MDSCs levels in blood were lowest in UNO-treated mice (Figure 6B), suggesting both a local and systemic reduction one day post-treatment. In contrast, blood M1 macrophages increased quickly after UNO treatment compared to control groups, and these higher levels were maintained on day 5, potentially reflecting specific systemic effects of nitric oxide on macrophage polarization (Figure 6C). Taken together, our findings suggest that UNO efficiently kills tumor cells in vivo and activates a favorable immune response.

### 3.3. Combining UNO with Anti-mPD-1 Reduces CT26 Primary Tumor Growth In Vivo

**Figure 7:**
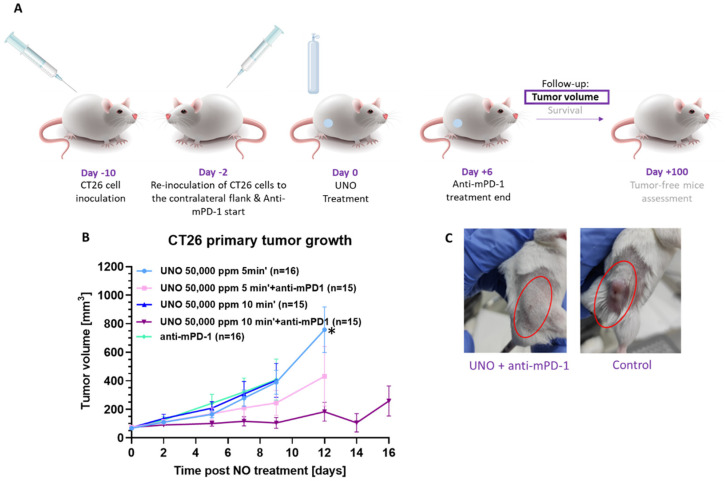
The local effect of UNO and anti-mPD-1 on CT26 tumor-bearing mice. (**A**) Study design. (**B**) Tumor growth curves of CT26 tumor-bearing mice (average tumor volume on treatment day 71.91 ± 37.24 mm^3^) treated with 50,000 ppm gNO for 5 or 10 min. Anti-mPD-1 dosing started two days before UNO treatment. An overall cumulative dose of 25 mg/kg over nine days of anti-mPD-1 was administered. Analysis via mixed model repeated measures (MMRM) with fixed effects for baseline tumor volume, study day, and treatment by study day interaction, * *p* = 0.0005 (at day nine post-UNO treatment). (**C**) Representative images of the primary tumor after treatment.

Our in vitro findings showing that UNO leads to the upregulation of PD-L1 on CT26 cells suggest that these cells might now be more susceptible to the effects of anti-mPD-L1 or anti-mPD-1 immune checkpoint inhibitors. Therefore, we performed in vivo testing combining UNO with anti-mPD-1 antibody treatments. CT26 cells were injected into the flanks of immunocompetent mice. When tumors reached a size of 50–100 mm^3^, tumors were intratumorally injected with 50,000 ppm UNO (*n* = 15–16 for each group) for 5 or 10 min, and tumor size was monitored (Figure 7A). Anti-mPD-1 dosing started two days before gNO treatment. As controls, mice were treated with each therapy alone. Mice treated with 5 or 10 min of UNO in combination with anti-mPD-1 experienced reduced tumor growth compared to mice treated with each treatment alone. The most dramatic effect was in mice treated with 50,000 ppm for 10 min + anti-mPD-1, in which the average tumor volume was significantly smaller than in mice treated with anti-mPD-1 alone nine days post-treatment (Figure 7B,C, *p* = 0.0005).

### 3.4. Increased Tumor Eradication Following UNO Intratumoral Treatment Combined with Systemic Anti-mPD-1 Administration In Vivo

**Figure 8:**
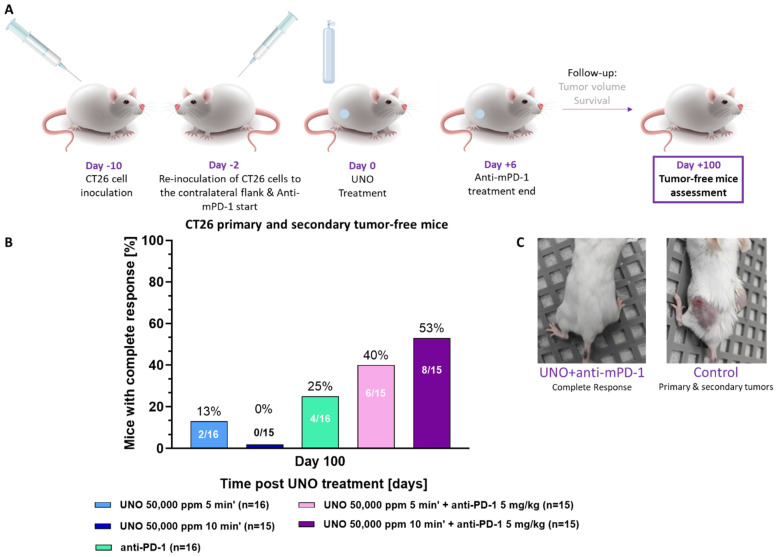
CT26 primary and secondary tumor-free mice. (**A**) Study design. (**B**) Percentage of primary and secondary tumor-free mice 100 days post-UNO treatment. Statistical analysis: Fisher’s exact test: *p* = 0.1489, pairwise treatment group comparison—50,000 ppm, 10 min + anti-mPD-1 vs. anti-mPD-1. (**C**) Representative images of 10 min UNO- + anti-mPD-1-treated mouse (**left**) vs. control mouse (**right**).

In addition to the significant short-term local effect of UNO treatment on the primary tumor, UNO treatment reduced the growth of both primary and secondary tumors for up to 100 days. Two days before the 50,000 ppm UNO treatment of the primary tumor, a second CT26 cell inoculation was applied to the contralateral flank and anti-mPD-1 treatment was initiated (Figure 8A). Importantly, the secondary tumor was induced before UNO treatment, allowing testing of the UNO and anti-mPD-1 combination for a potential abscopal effect.

Primary tumor regression was observed in 53% of the UNO- and anti-mPD-1-treated mice. Furthermore, these mice were also free of secondary tumors, an effect that was maintained for up to 100 days post-UNO treatment (Figure 8B,C).

### 3.5. Mice Survival Is Substantially Prolonged When Treated with 10 Min UNO and Anti-mPD-1 up to 100 Days Post-Treatment

**Figure 9:**
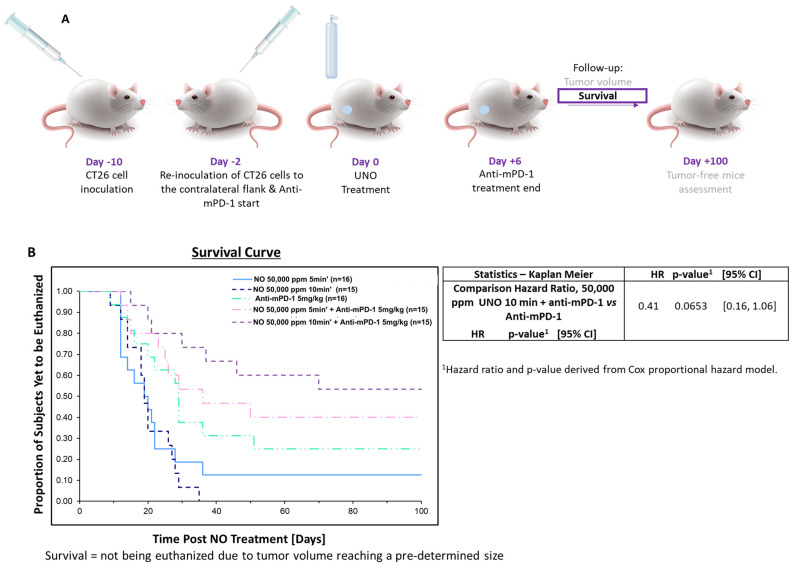
The effect of UNO and anti-mPD-1 treatment on mice survival. (**A**) Study design. (**B**) Survival curve, presented as a Kaplan–Meier curve. *p* = 0.065 for NO + anti-mPD-1 vs. anti-mPD-1.

Mice survival was monitored for 100 days post-UNO treatment. Life expectancy was considerably prolonged in UNO- + anti-mPD-1-treated mice compared to those treated with anti-mPD-1 (*p* = 0.065, Figure 9B).

## 4. Discussion

Although immunotherapy has emerged as a major therapeutic modality in cancer, many patients do not benefit from these treatments. While immune checkpoint inhibitors (ICI), which reactivate dysfunctional and/or exhausted T-cells, have marked efficacy against a broad range of cancers, 50–80% of patients with tumors for which ICIs are indicated do not benefit from these drugs, and many experience severe adverse events [33].

Our previous work presented a new tumor ablation method utilizing ultra-high concentrated gaseous NO (UNO). This treatment induces a strong and potent antitumor response by generating cancer antigens and exposing them to the patient’s immune cells. Our current study shows that UNO induces considerable cell death in vitro and in vivo, which is expected to promote neoantigen release [30]. In addition, our previous findings demonstrated that local administration of UNO promotes dendritic cell infiltration of the treated tumor, leading to increased tumor and systemic levels of several adaptive immune cells, including T-cells and B-cells, together with a decrease in splenic myeloid-derived suppressor cells (MDSCs). This NO-induced immune response significantly reduced challenge tumor formation and improved mice survival, suggesting that UNO ablation has therapeutic potential as an immunomodulating agent [31].

In this paper, we have shown an acute reduction in immunosuppressive T-regs in tumors treated with UNO, followed by an increase in cytotoxic CD8+ T-cells in a subset of tumors that responded to UNO. This implies that UNO treatment reduces immunosuppression and creates a transient favorable microenvironment that can contribute to immune potentiation. The coinciding cellular death observed in treated tumors may contribute to antigen release and further stimulate the immune response. A favorable tumor immune microenvironment may underlie the synergy observed in animals treated with anti-mPD1 in combination with UNO. We also noted a prolonged increase in M1 macrophages in UNO-treated animals, which may point to a systemic shift to a more pro-inflammatory antitumor immune profile.

We also used Annexin V and PI double-staining techniques to further explore the cell death mechanism after exposing tumor cells to 25,000–100,000 ppm NO for 10–60 s. The outcomes of this study showed that tumor cell death is dose- and time-dependent. The dominant status of tumor cells 24 h after exposure to 25,000 ppm NO for at least 1 min or 50,000 ppm for at least 30 s is late apoptosis. Analysis shows that the non-apoptotic/non-necrotic or early apoptotic CT26 cells upregulate PD-L1 expression on their surface in a time- and dose-dependent process. PD-L1 is statistically upregulated after exposure to at least 25,000 ppm for at least 1 min or 100,000 ppm NO for at least 10 s and may underly the in vivo effect. Furthermore, we showed that in vivo, 53% of the 10 min UNO and anti-mPD-1 group were primary and secondary tumor-free mice at day 100 post-UNO treatment. This observed synergy may be due to NO sensitizing CT26 cells to ICI therapy.

Based on our previous data showing T-cell penetration into the treated tumor mass and systemic upregulation [31], anti-mPD-1 was added to UNO to determine whether we could further augment the T-cell response. We induced secondary tumors before UNO treatment, in contrast to our previous work in which we induced the secondary tumors 21 days post-NO treatment. We evaluated the therapeutic efficacy of local UNO treatment combined with the systemic administration of the immune checkpoint inhibitor, anti-mPD-1, in a series of assays. The combination was significantly superior to either treatment alone, as seen by its effect on the treated primary tumor, total tumor burden, and survival. UNO administered for 10 min combined with anti-mPD-1 injections resulted in over half of the mice being primary and secondary tumor-free 100 days post-UNO treatment. While the CT26 model is considered a “hot” tumor type inducing more tumor-infiltrating immune cells than several other tumor models [17], anti-mPD-1 alone was notably inferior to the combination of UNO and anti-mPD-1 treatment.

In this paper, we demonstrate the potential for UNO to sensitize cancer cells to ICI therapy, thereby improving their response to an anti-mPD-1 antibody in vivo.

## 5. Conclusions

UNO treatment with primary tumor resection activates dendritic cells, which present cancer antigens to adaptive immune cells, significantly increasing the rejection of secondary tumors. PD-1 blockade, in combination with UNO, results in a significant increase in the proportion of mice that show primary tumor regression, a substantial increase in the rejection of secondary tumors, and a prolonged 100-day survival.

UNO induces a strong cellular response that appears to overcome anti-PD-1 resistance. Thus, combining UNO and immune checkpoint inhibitors, such as anti-PD-1, can have important clinical implications.

## Data Availability

The datasets used and/or analyzed during the current study are available from the corresponding author upon reasonable request.

## Abbreviations

ACK	Ammonium–chloride–potassium
ATCC	American Type Culture Collection
CTLA-4	Cytotoxic T-lymphocyte-associated protein 4
DAMPs	Damage-associated molecule patterns
ICI	Immune checkpoint inhibitors
ICD	Immunogenic cell death
gNO	Gaseous nitric oxide
i.p.	Intraperitoneal
IACUC	Institutional Animal Care and Use Committee
LPM	Liters per minute
MDSCs	Myeloid-derived suppressor cells
mPD-1	Mouse programmed cell death protein 1
MMRM	Mixed model repeated measures
MSI	Microsatellite instability
MMR	Mutations in mismatch repair
NOS	Nitric oxide synthase
PD-1	Programmed cell death protein 1
PD-L1	Programmed death ligand 1
PPM	Parts per million
PTFE	Polytetrafluoroethylene
PVC	Polyvinyl chloride
S.C.	Subcutaneous
UNO	Ultra-high-concentration gaseous nitric oxide

## Appendix A

Technical details:

In vivo UNO delivery:

Before each treatment, mice were anesthetized by an intraperitoneal (i.p.) injection of 100 mg/kg ketamine hydrochloride (Zoetis) and 10–20 mg/kg xylazine hydrochloride solution (Abic). Ten minutes later, mice were treated with intratumoral delivery of 50,000 ppm UNO. A pressure regulator was connected to the gas cylinder to allow an outlet pressure of approximately 2 bar. A stainless steel PTFE-coated hose was then connected to the pressure regulator and a manual flow meter. A PVC hose was connected to the flow controller, allowing a 23G hypodermic needle to be inserted into the approximate center of the tumor (about half of the tumor diameter, depending on the tumor size and shape) for administration of 50,000 ppm UNO at a 0.2 LPM rate for 5 or 10 min. There was no toxicity associated with UNO treatment.

### Appendix A.1. Flow Cytometry Markers for Cell Viability and Cell Subset Analysis

Myeloid cell profiling was performed using the following: APC-Vio770 anti-mCD45 (Miltenyi), PE anti-mCD11b (Miltenyi), PE-Vio770 anti-mF4/80 (Miltenyi), APC-anti-mCD11c (Miltenyi), with damping channels for Percp-Vio770 anti-mCD3 (Miltenyi), Superbright600 anti-mCD163 (Thermo Fisher Scientific), BV650 anti-mNK1.1 (BioLegend), VioBlue anti-mLy6g (Miltenyi), and FITC anti-mLy6c (Miltenyi). Ghost Dye 710 was used for dead cell exclusion.

M1 macrophages were defined as CD45^+^, CD11b^+^, F4/80^+^, CD11c^+^, CD163^−^, CD3^−^, NK1.1^−^, Ly6g^−^, and Ly6c^−^ cells.

Mononuclear MDSCs were defined as CD45^+^, CD11b^+^, CD3^−^, NK1.1^−^, Ly6g^−^, and Ly6c^high^ cells.

T-cell profiling was performed using the following: Percp-Vio770 anti-mCD3 (Miltenyi), APC/Fire 810 anti-mCD4 (BioLegend), BV650 anti-mCD25 (BD Biosciences), and BV421 anti-mFoxp3 (BD Biosciences) with a damping channel for Spark 574 anti-mCD8 (BioLegend). Zombie NIR was used for dead cell exclusion.

CD8 T-cells were defined as CD3^+^, CD4^−^, and CD8^+^ cells.

Tregs were defined as CD3^+^, CD4^+^, CD25^+^, Foxp3^+^, and CD8^−^, cells.

For each marker, fluorescence minus one (FMO) controls were used to determine the gating cutoff.

Cell viability in vivo was determined by Ghost Dye 710 positive staining in tumor samples.

### Appendix A.2. Tumor Volume Calculation

Local tumor growth was determined by measuring three mutually orthogonal tumor dimensions 2–3 times per week, according to the following formula:

$$\mathrm{Tumor}\;\mathrm{Volume}=\frac{\pi }{6}\times (\mathrm{Diameter}\;1\times \mathrm{Diameter}\;2\times \mathrm{Diameter}\;3)$$

### Appendix A.3. Conditions for Terminating the Participation of a Particular Animal in the Experiment

Animals found in a moribund condition and animals showing severe pain and enduring signs of severe distress were humanely euthanized. The health conditions of the animals were assessed using mouse distress scoring:

1. Appearance: Normal—0, Coat staring, ocular, or nasal discharge—1, Piloerection—2, Hunched up—3.
2. Hydration status: Normal—0, Skin tents when pinched, quickly recovers—1, Skin tents when pinched, slowly recovers—2, Skin remains tented, indicating severe dehydration—3.
3. Natural behavior: Normal, active—0, Less mobile and alert—1, Isolated—2, Restless/shivering/very still—3.
4. Body weight: Comparable to controls—0, Weight loss of 0–10%—1, Weight loss of 10–20%, Weight loss over 20%—3.
5. Tumor volume (of all tumors): 500–1000 mm^3^—1; 1000–1500 mm^3^—2; >1500 mm^3^—Animals with a >1500 mm^3^ tumor were humanely euthanized immediately.

When the total score was ≥7, the mouse was assessed 1–2 times daily, and wet food was placed at the bottom of the cage. When the score reached 10, or the tumor burden exceeded 1500 mm^3^, the mouse was first anesthetized using a ketamine and xylazine anesthetic mix, as previously described, and then euthanized by cervical dislocation. When animals were euthanized for humane reasons or found dead, the time of death was recorded as precisely as possible. The conditions for animal sacrifice described above were reflected as the endpoint for the survival experiment.
